# Item Response Models for Rating Relational Data

**DOI:** 10.1017/psy.2025.10016

**Published:** 2025-06-30

**Authors:** Chih-Han Leng, Ulf Böckenholt, Hsuan-Wei Lee, Grace Yao

**Affiliations:** 1 Department of Psychology, National Taiwan University, Taipei, Taiwan (ROC); 2 Kellogg School of Management, Northwestern University, Evanston, IL, USA; 3 Department of Biostatistics and Health Data Science, Lehigh University, Bethlehem, PA, USA

**Keywords:** item response theory (IRT), latent space model, rating relational data, rating scale model (RSM), social networks

## Abstract

This article introduces item response models for rating relational data. The relational data are obtained via ratings of senders and receivers in a directed network. The proposed models allow comparisons of senders and receivers on a one-dimensional latent scale while accounting for unobserved homophilic relationships. We show that the approach effectively captures reciprocity and clustering phenomena in the relational data. We estimate model parameters using a Bayesian specification and utilize Markov Chain Monte Carlo methods to approximate the full conditional posterior distributions. Simulation studies demonstrate that model parameters can be recovered satisfactorily even when the dimensionality of the network is small. We also present an extensive empirical application to illustrate the usefulness of the proposed models for complete and incomplete networks.

## 1 Introduction

This article introduces rating scale models (RSMs) for analyzing rated networks built from relational data, specifically when each sender (i.e., individual) assigns ratings to one or more receivers in a directed network. In rated networks, nodes represent individuals, and edges encode relational ratings, such as social support (Best & Blakeslee, 2020; Ferligoj & Hlebec, 1999; Hlebec & Ferligoj, 2002), advice (De Lange et al., 2004), or friendliness (De Lange et al., 2004). Typically, such a scale has a small number of ordered response categories —for example, “close personal friend,” “friend,” “acquaintance,” “someone I have met,” “someone I have heard of but not met,” or “someone I have not heard of” (Freeman, 1984; Freeman & Freeman, 1979, 1980).

Five notable features characterize the proposed model framework. First, our approach accommodates tied ratings in contrast to many ranking models for network data that do not (Gormley & Murphy, 2007; Krivitsky & Butts, 2017; Sewell & Chen, 2015). Second, the proposed models account for the ordinal nature of the rating data and allow for distances between response categories to be arbitrarily different. In this regard, they extend the current toolset for the analysis of networks based on categorical (e.g., contact Dekker et al., 2022; Luo et al., 2022) and continuous scales (e.g., travel cost Liu et al., 2022 and transaction Najafi & Saghaei, 2021).

Third, the proposed models yield readily interpretable insights about a rated network. They capture both reciprocity in the dyadic ratings and unobserved interactions among the members of the social network introduced by homophily and transitivity. Thus, they facilitate comparing senders and receivers of a network while taking into account homophilic relationships. Since the models can be applied to both complete and incomplete networks, obtaining these insights is possible even when a substantial percentage of dyadic ratings are missing.

Fourth, the proposed approach allows for assessing model (mis)fit by focusing on identifying participants who systematically deviate from the specified model structure. Fifth, the models facilitate comparisons across multiple networks regarding their levels of reciprocity and clustering and the inclusion of covariates that may help explain differences between raters and receivers within as well as across networks.

The remainder of this article is organized as follows. The next section reviews the literature and situates our contributions in the broader context. Subsequently, we formally introduce the item response models and outline their core features. Model estimation is described in Section 3, which presents our Bayesian approach and details of the utilized Markov Chain Monte Carlo (MCMC) algorithm. A simulation study investigates parameter recovery in Section 4. We also provide model tests and study the relationships between model parameters and established reciprocity and clustering indices. An empirical application demonstrates the usefulness of the proposed approach in Section 5. The article concludes with a discussion and outlook for future research in Section 6.

### 1.1 Literature review

Current methods for modeling ordinal social network data have limitations that warrant further advancements. First, one common approach involves finding an optimal cut-off point or threshold to dichotomize the original ordinal scale (Arabie et al., 1978; Breiger et al., 1975; Doreian et al., 1996; Krackhardt & Handcock, 2007; Moody et al., 2005; Wasserman, 1980). However, such thresholding can be sensitive to how the cutoff is selected, resulting in information loss or biased estimates (Baggio, 2019). Hence, models that preserve the ordinal structure are often preferable.

Second, researchers frequently utilize exponential random graph models (ERGMs) to model the presence or absence of an edge between a sender and a receiver (Frank & Strauss, 1986; Pattison & Wasserman, 1999; Robins, 1999; Robins et al., 2007; Pattison & Wasserman, 1999; Snijders et al., 2006; Wasserman & Pattison, 1996). Building on the ERGM, Krivitsky and Butts (2017) developed a model for rank-order relational data that utilizes the generalized framework proposed by Krivitsky (2012). This approach converts the rankings of receivers from each sender into binary paired comparison matrices modeled with a binary ERGM. As a result, these models are suited for analyzing ranked networks and do not consider cross-rater comparisons. They are not applicable to rated network data since they cannot accommodate outcomes where senders assign the same rank to multiple receivers.

Third, a similar limitation applies to the Plackett-Luce model (Plackett, 1975) when applied to rank-order network data. In an analysis of voting data, Gormley and Murphy (2007) combined the Plackett-Luce model with Hoff et al. (2002)’s latent space model. This model can handle rank-order scales without requiring pre-processing and can facilitate comparisons between the locations of voters and candidates in a two-dimensional latent space. However, because this version of the Plackett-Luce considers only the preferences of voters for political candidates, it lacks reciprocity in its network structure when receivers rate senders. Although Sewell and Chen (2015) advanced the Plackett-Luce model to account for reciprocity between senders and receivers, this model is also not well suited for rating data. It cannot accommodate the common situation of senders or receivers expressing ties when using the same rating category multiple times.

Fourth, researchers also commonly use the ordinal logit/probit link functions to model ordinal data. For instance, Hoff (2021) proposed the Additive and Multiplicative Effects Network (AMEN) models to analyze the ordinal social network data, utilizing an ordinal probit link function in conjunction with a multiplicative effect via an inner product specification of the latent space model. The AMEN models leverage the latent space model to capture network dependencies within the ordinal probit link framework, enabling the analysis of ordinal social network data. This approach, however, neglects the varying distances among rating options, which can be helpful for interpretative accuracy.

## 2 Rating scale models for network data

Because of the limitations noted in the previous section, we propose two models and their special cases that are tailored to the analysis of relational rating data. Under the first model, referred to as Dyadic Relation RSM (DR.RSM), each individual in the network can send and/or receive ratings Building on Jeon et al. (2021)’s and Luo et al. (2023)’s latent space item response theory (IRT) models, we extend the DR.RSM to go beyond the dyadic relation between senders and receivers and account for local dependencies among individuals. Local dependencies are represented by distances among individuals in a low-dimensional unobserved metric space. We refer to this model as the Latent Space Rating Relation Model (LSRRM). The LSRRM compares senders and receivers on a one-dimensional latent scale while accounting for homophilic relationships. Even when only partial data are available, this model can estimate such essential network characteristics as reciprocity and clustering.

Let 
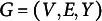
 be a finite rated network, with a non-empty set of individuals *V*, a set of relations *E*, and a function *Y*. Specifically, *V* is a set containing *N* individuals, that is, 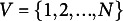 . Each relation 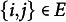 is formed by an ordered pair of individuals 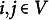 ; that is, 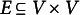 , also known as the sample space. The function 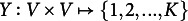 assigns a rating to each relation, which is the random variable we consider. Thus, the range of *Y* is defined as 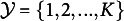 , representing a finite set of positive ordered integers, where 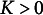 is the largest number. *Y* can also be interpreted as individuals’ responses to rate their relations on a *K*-point Likert-type scale. For a given rating, let 
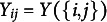
 denote the rating of individual *i* to individual *j*. Here, as it is common in network studies, the individual who sends ratings is referred to as the “sender,” while the individual who receives ratings is referred to as the “receiver.” Since senders do not rate themselves, 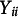 is undefined for all 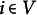 . A complete network is formed when every 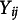 is observed for all pairs of 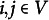 (i.e., 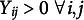 ).

The DR.RSM models 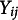 based on the person-specific parameters of sender *i* and receiver *j*, along with a set of network-specific threshold parameters. We denote by 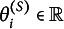 and 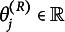 the parameters of sender *i* and receiver *j*, respectively, and by 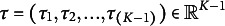 the threshold parameters. Specifically, we assume that sender and receiver parameters capture uni-dimensional latent traits (e.g., personality traits) that affect the levels of giving and receiving ratings, which are measured by their rating behaviors. Each element within the threshold parameters represents a gap that the senders must overcome when deciding to assign a higher rating value. Consequently, the probability of rating 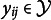 to receiver *j* for sender *i* can be specified as 
(1)

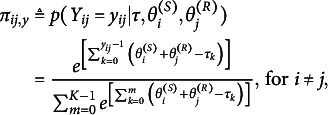

where 
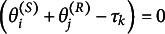
 if 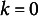 .

One special case of (1) is of interest. When the number of categories *K* is 2, that is, 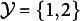 , the DR.RSM reduces to a form of the Rasch model, which we refer to as DR.Rasch. The DR.Rasch model shares similarities with the dyadically independent ERGM in modeling binary relationships; however, in contrast to the ERGM, it represents individuals’ dyadic relations on a one-dimensional latent scale.

Extending the DR.RSM, the LSRRM model includes a latent-space representation to capture local dependencies in the ratings (Jeon et al., 2021; Jin & Jeon, 2019; Kang et al., 2023; Luo et al., 2023). For this model part, it is assumed that (a) individuals are mapped onto the same *V*-dimensional unobserved metric space, (b) the distances among senders and receivers are independent of the sender and receiver parameters, and (c) the distances reduce the probability defined in equation (1) but control the decrease for similar individuals. Thus, the LSRRM captures such homophilic relations as the increased likelihood of individuals becoming friends with others who are similar to them. It also accounts for transitivity, which measures the degree to which connections are formed between the neighbors of a rater (Hoff et al., 2002). We specify the LSRRM as follows: 
(2)

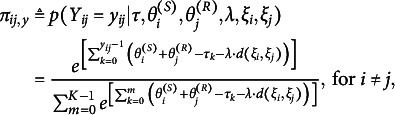

where 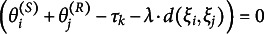 if 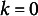 and 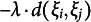 is the homophily term, which represents the interactions among individuals on the V-dimensional latent metric space. In the homophily term, 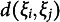 measures the distance between sender *i*’s and receiver *j*’s latent positions 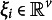 and 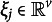 , which can be calculated using any distance function, such as the Euclidean distance ( 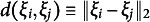 , Hoff et al., 2002), projection distance ( 
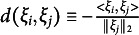
 , Hoff et al., 2002), and inner product distance ( 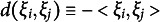 , Hoff, 2005). Furthermore, 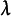 captures the degree to which homophilic relations are present in the data. When 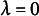 , equation (2) reverts to equation (1), the DR.RSM.

The selection of a distance function can be based on three considerations related to network characteristics, interpretability, and presence of asymmetrical relationships: (1) In regards to network characteristics, the Euclidean distance has been shown to effectively capture transitivity and community structures (Hoff, 2021; Hoff et al., 2002), while the projection and inner product distances are superior at handling clusterability induced by weak ties (e.g., acquaintance), which act as bridges between different components (Hoff, 2005; Hoff, 2021; Nowicki & Snijders, 2001); (2) In regards to interpretation, the Euclidean distance is a natural choice for Euclidean space, making it more aligned with social network analyses that use network positions for inference, and thus facilitates easier interpretation. In contrast, the projection and inner product distances are constructed in the vector space, which requires careful interpretation of vector directions and angles (Hoff, 2005; Jeon et al., 2021); (3) In regards to capturing asymmetrical relationships ( 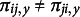 ), both projection and inner product distances are more effective than the Euclidean distance (Hoff et al., 2002). A comparison between the projection and inner product functions reveals that the projection distance is more effective in capturing the activity levels of senders (Hoff et al., 2002).

In the following presentation and in our empirical application, we employ the Euclidean distance for ease of interpretation and to illustrate individuals’ interactions in a latent space, similar to social network analysis. However, we also report the performance of these three approaches in fitting empirical data in Section 5. Code for estimating the projection distance and inner product distance versions of the LSRRM is available on GitHub.

We define the density function of 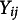 as 
(3)

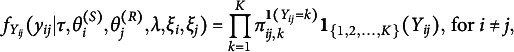

resulting in a categorical distribution with *K* components, where 
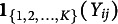
 is an indicator function that is 1 if 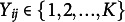 or 0 otherwise. Additionally, let 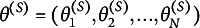 , 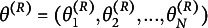 , 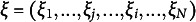 . We assume that the ratings of sender *i* to all receivers excluding *i* conditional on 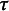 , 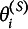 , 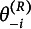 , 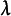 , and 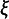 are independent (the subscript, “ 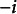 ,” denotes the exclusion of *i*). Similarly, the ratings received from all senders excluding *j* to receiver *j* given 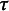 , 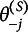 , 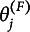 , 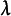 , and 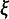 are also specified to be independent.

Additionally, we specify 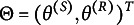 and 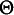 to follow a bivariate normal distribution: 
(4)

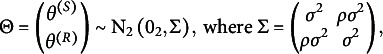

where 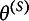 and 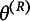 are assumed to have equal variance and to be correlated with the correlation coefficient 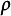 . Under this setup, 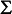 determines the degree of reciprocity. Specifically, 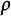 captures the similarity in the ratings between senders and receivers. The relationships are approximately symmetric when 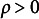 and are approximately asymmetric when 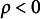 . The variance term 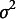 further moderates the degree of symmetry or asymmetry.

The latent positions, 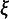 , are specified to follow a *V*-dimensional normal distribution, that is, 
(5)

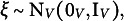

with a fixed 0 mean vector and identity covariance matrix (see also Jeon et al., 2021; Jin & Jeon, 2019; Kang et al., 2023; Luo et al., 2023). In our application, we set 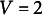 to facilitate visualizing the individuals’ interactions in the latent space.

One special case of (2) is of interest. When the number of categories *K* is 2, we obtain a latent-space version of the DR.Rasch model. This model extends the latent cluster random effects model (Krivitsky et al., 2009) by allowing for correlated sender and receiver parameters and letting 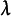 differ from 1. As a result, the latent-space DR.Rasch model provides more flexibility in modeling reciprocity and clustering in binary social network data.

## 3 Estimation procedure

All the parameters in the LSRRM are treated as random effects and estimated using the Hamiltonian Monte Carlo (HMC) method implemented in the “nimble” R package (Turek et al., 2024). We outline how to use the nimble package for estimating a version of the LSRRM model in Appendix A.1. A parallel estimation version is available on GitHub.

The priors for the DR.RSM model part are specified as, 
(6)

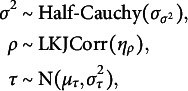

where LKJCorr is the Lewandowski–Kurowicka–Joe (LKJ) correlation prior, and 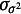 and 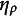 are set as 2.5 and 1, respectively, following the studies of Kang et al. (2023); Lewandowski et al. (2009); Stan Development Team (2020). In addition, we set 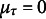 and 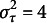 , as in Cho and Cohen (2010), Cohen and Bolt (2005), Huang (2016), Huang et al. (2013), Jin and Wang (2014), and Li et al. (2006).

For the homophily term, we specify 
(7)

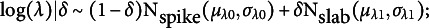

that is a spike-and-slab prior (Ishwaran & Rao, 2005; Mitchell & Beauchamp, 1988) is assigned to 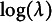 , which is used to examine if there is a conditional dependence among senders and receivers (Jeon et al., 2021; Kang et al., 2023).

Identically to the setup of Kang et al. (2023), for the spike part, given 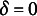 , we specify 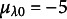 and 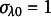 , which defines the situation when conditional dependence does not exist. Thus, 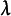 can be shrunk to 0, and the LSRRM is reduced to the DR.RSM. In contrast, given 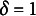 , for the slab part, we have 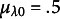 and 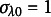 , so 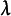 can be estimated with an uninformative or weakly informative prior if conditional dependence exists.

In determining 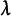 , we further specify 
(8)

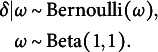

Hence, 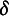 can be determined by 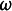 , estimated with a weakly informative prior. Therefore, if the estimate of 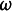 is greater than .5, the LSRRM is selected (Jeon et al., 2021).

Because the distances between senders and receivers remain unchanged when reflected, rotated, or translated, we can identify only the distances between individuals but not their positions in the latent space. To address this issue, we post-process the posterior sample of 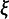 by using Procrustes matching to align each of the posterior samples of 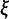 with the reference set, which consists of the latent positions having the highest log posterior density value (Jeon et al., 2021; Jin & Jeon, 2019; Kang et al., 2023; Luo et al., 2023; Shortreed et al., 2006).

**Figure 1:**
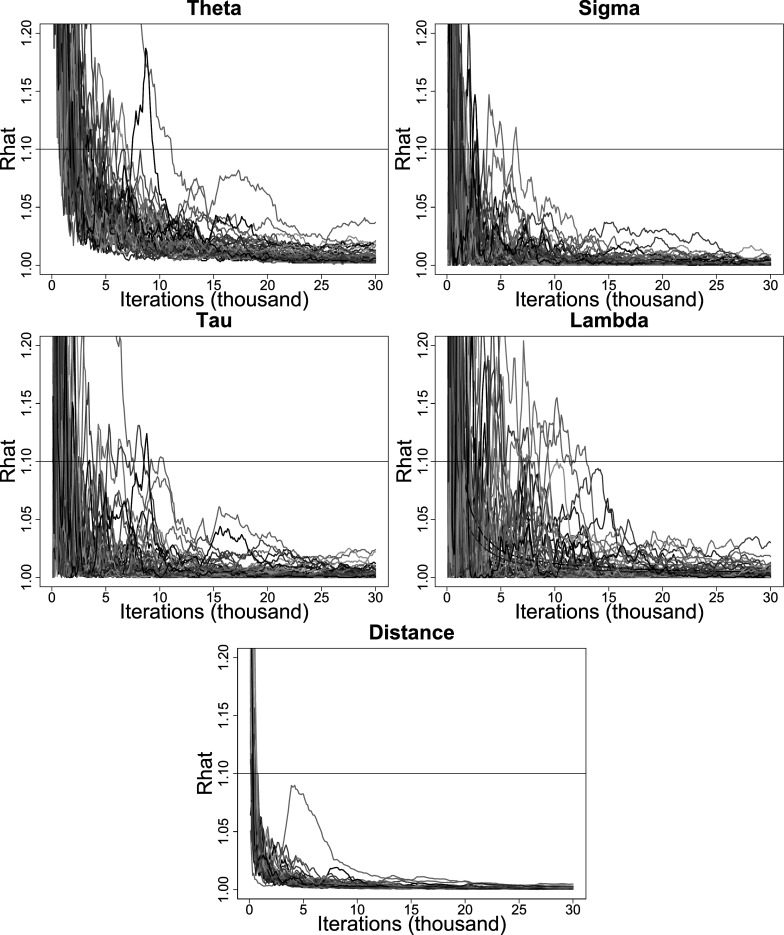
Potential scale reduction statistics ( 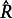 ).*Note:* 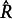 s are calculated every 100 iterations and plotted on the *y*-axis against the number of iterations on the *x*-axis. Each line illustrates the changes in the average 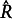 for each parameter in a replication.

## 4 Simulation studies

### 4.1 Overview of studies

This section reports four studies to evaluate the recovery ability of the LSRRM for (a) model parameters, (b) reciprocity, and (c) clustering and to assess the (d) clusterability of the LSRRM parameters. For all conditions in studies (a)–(c), three sample sizes were specified with 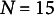 , 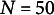 , and 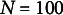 . By selecting a small, medium, and large sample size, we can assess the lower limit of precision by which the LSRRM parameters can be estimated as well as improvements in precision when a sample size increases. Because studies (a)–(c) focus on precision, for study (d), only one sample size, 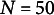 , was specified. This study evaluates the LSRRM’s performance in capturing the clusterability of the individuals’ latent interactions. We report the accuracy in estimating the distances among individuals instead of their positions in the latent space for identifiability reasons. We fixed the response scale for all studies to have five categories with equally spaced thresholds.

In studies (b) and (c), we assess the model’s performance in estimating a network’s reciprocity and clustering characteristics. As mentioned in Section 1, a network is constructed by individuals and their connections. We can investigate dyads to understand how two individuals influence or interact with each other by using the reciprocity index, which measures the likelihood of mutual connections between them (Garlaschelli & Loffredo, 2004). However, the fundamental components of social networks are triads, not dyads (Holland & Leinhardt, 1971; Wasserman & Faust, 1994) because the impact of one link onto another can only be explored within triads. For instance, the clustering index measures the likelihood of connecting the neighbors of one individual. This index assesses local clustering of each individual and global clustering by averaging the local terms across all individuals (Boccaletti et al., 2006). Another type of global clustering, known as transitivity, calculates the ratio of “the number of triangles (where three individuals are fully connected)” to “the number of triples (where three individuals are connected by at least two links)” (Costa et al., 2007). Hence, clustering indices generally reflect how interconnected the neighbors of each individual are (Iacobello et al., 2021). We examine the extent to which the DR.RSM and the latent-space model parts can capture reciprocity and clustering properties of a network, respectively.

In study (d), we consider scenarios where similar individuals are densely grouped in the latent space and form communities. These communities are assumed to represent how individuals interact. Individuals may interact based on observed characteristics (e.g., gender, friendship, and neighborhood) as well as unobserved characteristics (e.g., latent traits). We assess how well the LSRRM can capture these interactions among individuals in the latent space.

To assess the convergence behavior of the algorithm, we generated 50 datasets with a sample size of 15 using the LSRRM. We then fit these dataset to the LSRRM to evaluate convergence based on the potential scale reduction factor 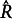 (Brooks & Gelman, 1998) with the criterion 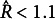 .

Each dataset was generated under the following specifications: 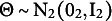 , 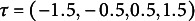 , 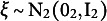 , and 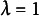 . We calculated 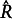 per 100 iterations and took the average for each parameter. A plot of the changes of each parameter’s 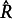 s in Figure 1 shows that most parameters converged before 10,000 iterations, and few converged within 15,000 iterations. Based on these results, we ran three independent chains for each replication in the simulation studies. For each chain, the total number of iterations was set to 60,000. The first 20,000 iterations were discarded as a burn-in regime, and an interval of 40 iterations was used to thin the remaining iterations.

### 4.2 Estimation accuracy of model parameters

**Figure 2:**
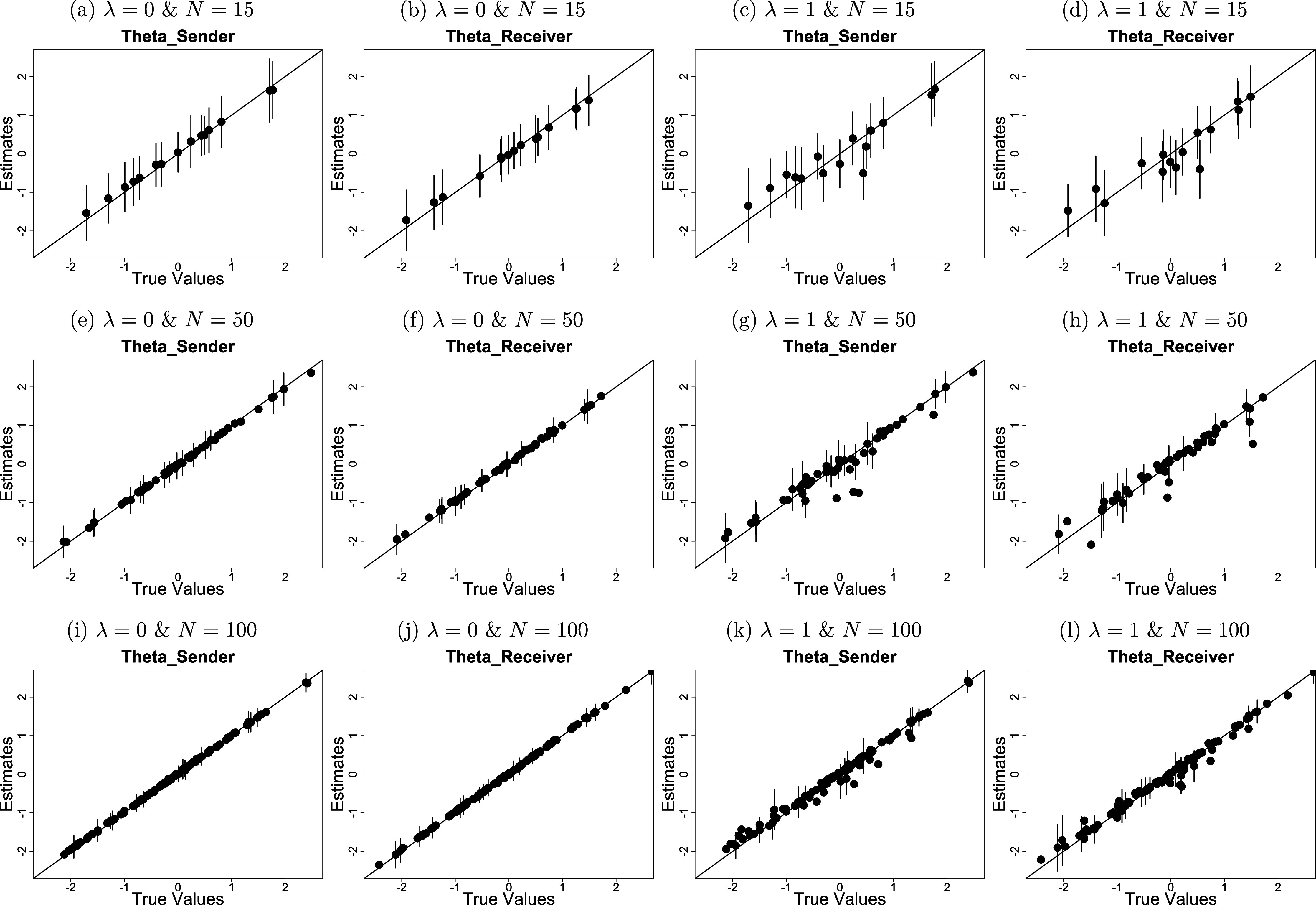
The estimates of 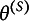 and 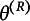 .*Note:* Subfigures (a)(b)(e)(f)(i)(j) and (c)(d)(g)(h)(k)(l) display the case of 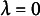 and 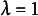 , respectively. Subfigures (a)(c)(e)(g)(i)(k) and (b)(d)(f)(h)(j)(l) display the estimates of 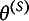 and 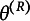 , respectively. Subfigures (a)–(d), (c)–(h), and (i)–(l) display the case of 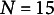 , 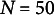 , and 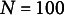 , respectively.

In study (a), we generated 50 datasets under the LSRRM to assess the estimation accuracy of the model parameters for the three sample sizes 15, 50, and 100. We used the same true values as in the convergence study reported above. We also set 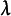 equal 0 and 1 to test whether 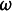 can diagnose conditional dependence. The bias, root mean square error (RMSE), and frequentist coverage probability (CP) of the EAP estimates were used to evaluate estimation performance. The CP value measures the percentage of the true value falling within the 95% highest posterior density (HPD) intervals (Chen & Shao, 1999) of the posterior samples across all 50 replications. However, we do not compute the CP value for the 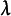 estimate when 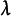 is set to zero because its true value will not fall within a range that begins with a non-zero positive number. Also, we do not calculate the CP values for the latent distances among individuals, as these distances are not considered model parameters. Results in the form of averages are reported in Table 1. Figure 2 displays a scatterplot of the average estimates of 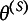 and 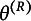 and their 95% confidence interval versus their true values.

**Table 1:** Average bias, RMSE, and CP values for the estimates of the LSRRM model parameters

	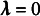								
Parameter	*N*=15			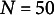			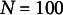		
	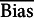	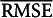	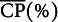	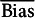	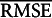	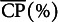	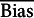	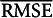	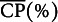
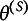	.0539	.3331	97.47	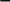 .0157	.1735	98.08	.0160	.1236	97.40
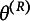	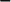 .0083	.3104	97.87	.0379	.1755	96.96	.0173	.1222	97.56
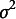	.1923	.3655	100.00	.0430	.0913	100.00	.0342	.0447	100.00
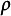	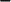 .0352	.1013	100.00	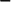 .0054	.0365	100.00	.0070	.0193	100.00
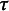	.0000	.2624	95.50	.0000	.0758	93.00	.0000	.0353	95.19
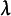	.0108	.0109		.0103	.0103		.0140	.0140	
	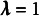								
Parameter	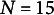			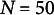			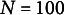		
	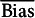	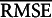	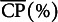	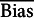	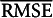	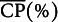	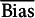	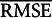	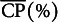
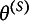	.0024	.4837	97.14	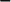 .0516	.3223	94.07	.0171	.2239	96.43
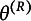	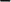 .0597	.4766	98.57	.0019	.3124	95.07	.0148	.2241	96.10
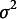	.3397	.4975	100.00	.0999	.1333	100.00	.0252	.0548	100.00
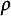	.0403	.1168	100.00	.0158	.0579	100.00	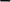 .0103	.0393	100.00
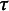	.0000	.3525	97.32	.0000	.0922	95.37	.0000	.0516	94.12
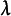	.0621	.1622	100.00	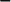 .0145	.0531	98.15	.0178	.0366	100.00
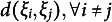	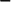 .0717	.5968		.0083	.4742		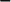 .0024	.3219	

*Note*: 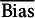 : Average bias. 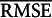 : Average RMSE. 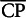 : Average CP.

When 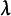 is set to zero for 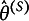 and 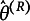 , the average biases are 0.0539 and 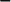 0.0083, respectively, for a sample size of 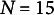 . The parameter estimates are highly correlated with their true values, with a correlation coefficient close to 1, as shown in subfigures (a) and (b) of Figure 2. The average RMSE values are 0.3331 and 0.3104. These values decrease significantly when the sample size increases [ 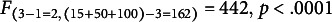 ] and [ 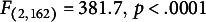 ]. The average CP values exceed 95% and do not change significantly as the sample size increases [ 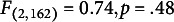 ] and [ 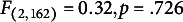 ]. The average estimates of 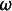 and 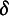 over the 50 replications are 0.3469 and 0.0429, respectively. Since 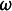 is less than 0.5 and 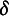 is around 0, 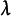 is estimated to be 0 with an average bias of 0.0108 and an average RMSE of 0.0109 for 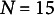 .

When 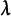 is set to one, the average biases for 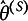 and 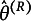 are 0.0024 and 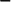 0.0597, respectively, for a sample size of 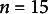 . The correlations between the parameter estimates and true values are 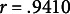 and 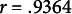 and are relatively more scattered compared to the case of 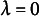 , as depicted in subfigures (c) and (d) in Figure 2. The average RMSE values are 0.4837 and 0.4766, which are significantly greater than those in the case of 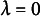 [ 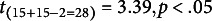 ] and [ 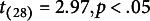 ], but are significantly improved as the sample increases [ 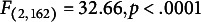 ] and [ 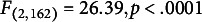 ]. The average CP values are around 95% and remain stable for the considered sample sizes [ 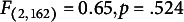 ] and [ 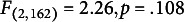 ].

The average estimates of 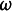 and 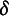 over 50 replications are 0.6650 and 0.9975, respectively. Since 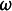 is greater than .5 and 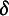 is around 1, 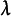 is estimated to be greater than zero and approximately one with an average bias of 0.0621, an average RMSE of .1622, and an average CP close to 100% for 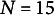 . Consequently, there is support for the conditional dependence specification. The distances between latent positions are estimated with an average bias of 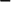 0.0717 and an average RMSE of 0.5968. The average RMSE decreases when the sample size increases [ 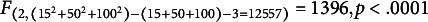 ].

### 4.3 Recovery of the reciprocity index

Studies (b) and (c) examined the model’s ability to estimate reciprocity and clustering characteristics of the data. In study (b), we calculated the reciprocity index using the method proposed by Squartini et al. (2013) for weighted and directed network structures; that is 

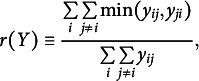

where 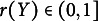 , measures the degree to which any two individuals rate each other identically in a network. In a complete network where every rating is above zero, the index will approach 0 if the differences in the ratings between pairs of individuals approach infinity. Conversely, the index is equal 1 if all individuals rate each other identically. Given the settings of this study, the lower bound of the reciprocity index is 0.33 for an extreme case where the elements of the upper and lower triangular matrix of *Y* are 5 and 1, respectively.

We examined the model’s ability to recover the reciprocity index by varying the parameters 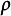 and 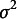 . These parameters determine the degree of symmetry in the ratings by the sender and receivers (see equation (4)). A numerical example in Appendix B.1 illustrates that 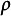 and 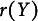 are highly correlated. The size of the correlation coefficient is weakly moderated by 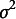 , demonstrating that this parameter also captures the network’s reciprocity behavior to some extent.

Three levels of reciprocity were tested. For each level, 50 datasets were simulated using the LSRRM. To vary the level of reciprocity, we specified the distribution of 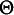 with 
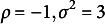
 for the lower level; 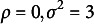 for the middle level; and 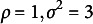 for the higher level. The other parameters were set as 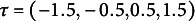 , 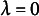 , and 
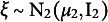
 for each dataset.

For the three sample sizes of 15, 50, and 100, the mean (M) and standard deviation (SD) of the reciprocity index values for the simulated data are approximately 0.55 and 0.02, respectively, at the lower level; 0.70 and 0.03, respectively, at the middle level; and 0.90 and 0.02, respectively, at the higher level. The LSRRM was fit to each dataset and the model estimates were used to simulate a new dataset. This allowed us to assess the recovery ability of the LSRRM for the reciprocity index by comparing the reciprocity indices between the generated and the simulated datasets. The recovery ability was evaluated using bias and RMSE, and the results are summarized in Table 2.

**Table 2:** Average bias and RMSE values for the estimated reciprocity index

	Reciprocity								
	Lower level			Middle level			Higher level		
Sample size	15	50	100	15	50	100	15	50	100
Bias	.0014	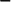 .0003	.0004	.0063	.0006	.0013	.0003	.0006	.0006
RMSE	.0161	.0052	.0027	.0168	.0049	.0031	.0139	.0035	.0026

As shown in Table 2, the model’s ability to recover the three levels of the reciprocity index was similar when *N* = 15, with bias values close to 0 and RMSE values ranging from 0.0139 to 0.0168. Additionally, the RMSE values decreased as the sample size increased to 50 and 100. We also simulated the case of 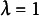 for 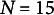 . The bias and RMSE values were 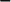 0.0074, 0.0215 at the lower level 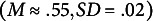 , 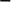 0.0014, 0.0225 at the middle level 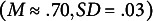 , and 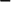 0.0004, 0.0190 at the higher level 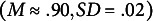 . These results indicate that the value of 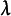 does not affect the precision in estimating the reciprocity index. Furthermore, the results for the two 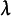 cases are similar, although the RMSE values when 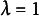 are slightly greater than when 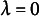 .

### 4.4 Recovery of the clustering index

In study (c), the clustering index was calculated using McAssey and Bijma (2015)’s method for weighted and directed completed networks, which is specified as 

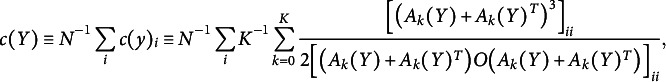

where 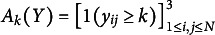 and *O* is a matrix consisting of zeros in the diagonal and ones in all other positions.

The index 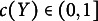 represents the average likelihood that each individual’s strong neighbors are strong neighbors with one another. The value of 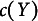 approaches 0 when all ratings are one and *K* is infinitely large. In our case, the lower bound of 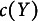 is 0.2. Conversely, 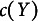 equals 1 when all ratings are equal to *K*. Within the numerator of 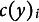 , the cubic term determines the number of *k*-level ratings passed among any pairs connected with the individuals *h*, *i*, and *j*, resulting in the number of observed directed triangles involving individual *i*. Furthermore, the denominator quantifies how many pairs of individuals *h* and *j* give/receive *k*-level ratings to/from individual *i*, leading to the total number of directed triangles involving individual *i*. Hence, 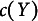 also evaluates transitivity.

We assessed LSRRM’s ability to recover the clustering index by adjusting the model parameter 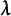 , as we expected that this parameter moderates the level of homophily and transitivity as shown in equation (2). A numerical example in Appendix B.2 illustrates the negative strong relationship between 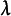 and 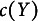 . This example demonstrates that the homophily term effectively captures the network’s clustering characteristic with negative 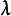 capturing the degree of dependency among individuals.

Three levels of clustering were examined. For each level, we generated 50 datasets using the LSRRM. Three levels of the clustering index were considered by setting 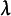 equal 0.1 for the higher level, 1 for the middle level, and 3 for the lower level. The other parameters were generated as follows: 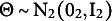 , 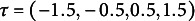 , and 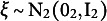 for each dataset.

For the sample size of 15, the mean and SD of the clustering index values for the simulated data are approximately 0.25 and 0.04, respectively, at the lower level; 0.45 and 0.03, respectively, at the middle level; and 0.65 and 0.05, respectively, at the higher level. For the sample size of 50, the mean and SD of the clustering index values for simulated data are approximately 0.35 and 0.03, respectively, at the lower level; 0.50 and 0.01, respectively, at the middle level; and 0.60 and 0.02, respectively, at the higher level.

Similar to study (b), each dataset was fit by the LSRRM and the estimated model parameters were used to simulate a new dataset. We evaluated the model’s ability to recover clustering by comparing the clustering indices of the generated and simulated datasets. We calculated bias and RMSE to assess the recovery ability of the model. Results are summarized in Table 3.

**Table 3:** Average bias and RMSE values for the estimated clustering index

	Clustering								
	Lower level			Middle level			Higher level		
Sample size	15	50	100	15	50	100	15	50	100
Bias	.1303	.0252	.0095	.0663	.0190	.0076	.0142	.0034	.0031
RMSE	.1370	.0316	.0151	.0798	.0241	.0134	.0321	.0101	.0065

Table 3 shows that most indices were overestimated, with bias values ranging from 0.0142 to 0.1303 for 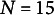 with higher index levels exhibiting lower bias. The recovery of the three levels of the clustering index improved as the index level increased, with RMSE values changing from 0.1370 to 0.0321. Recovery improved further with a sample size of 50 and 100. These findings suggest that the ability to recover clustering is sensitive to 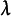 and latent distances; however, estimation accuracy improves when the sample size increases.

In summary, when 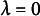 , in studies (a) and (b), LSRRM performed well in estimating model parameters for the considered sample sizes of 15, 50, and 100. This is also reflected by its ability to recover the reciprocity of a network. When 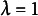 , studies (a)–(c) demonstrate satisfactory performance in recovering the model parameters, the reciprocity index, and the clustering index. Further improvements are observed for the larger sample sizes.

### 4.5 Clusterability

Study (d) evaluated the model’s capacity to capture individuals’ latent interactions. Two designs were utilized: (1) two equally sized components and (2) three unequally sized components. For design (1), we assumed that the first 25 individuals and the remaining 25 individuals form cliques within the network. For design (2), we assumed that the first 25 individuals, the next 15 individuals, and the remaining 10 individuals form cliques within the network.

**Figure 3:**
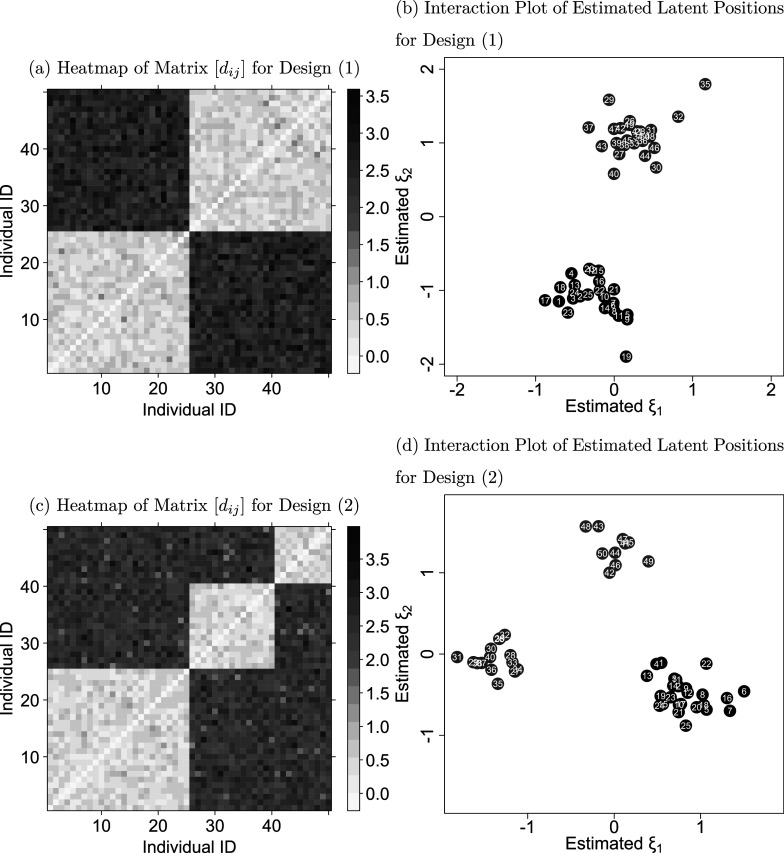
Examples of distance matrices and estimated latent positions for designs (1) and (2).*Note:* Subfigures (a) and (c) display heatmaps of the distance matrix 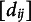 for designs (1) and (2), where a darker color indicates a larger distance. Subfigures (b) and (d) display the corresponding interaction plots of the latent positions estimated by the LSRRM.

For each design, we first simulated a distance matrix ( 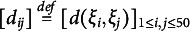 ), as shown in subfigures (a) and (c) of Figure 3 for designs (1) and (2), respectively, where a darker color represents a longer distance between two individuals. Specifically, following the study of Kang et al. (2023), we assigned short distances from 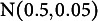 to the within-group distances. To represent the between-group distances, we assigned larger distances using 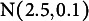 . Since the distance matrix is symmetric, we set 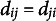 for all pairs of *i* and *j*. Subsequently, we generated 50 datasets using the LSRRM, with specified distance matrices and parameters: 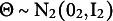 , 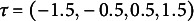 , and 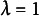 . For each dataset, after fitting the LSRRM, we submitted the estimates of 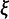 to a K-means analysis. We determined the optimal number of clusters using Silhouette scores. To evaluate the models’ ability to separate individuals, we calculated accuracy as the ratio of correctly assigned individuals to their corresponding cliques.

For design (1), the average accuracy across the 50 datasets is 100%. The CP value is 100%, with the average RMSE values for 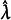 and distance estimates of 0.0587 and 0.4414, respectively. These values are closely aligned with the results from study (a). For design (2), both the average accuracy and CP value remain at 100%. The average RMSE values for 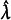 and distance estimates are 0.0725 and 0.4312, respectively. The RMSE value for 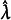 is slightly higher than the one observed in study (a), while the RMSE for the distance estimates is comparable to that of study (a). These results show that the LSRRM is well-suited to effectively recover unobserved cliques in rating relational data.

## 5 Empirical study

In this section, we illustrate the effectiveness of the LSRRM using network data obtained from senior high school students in Taiwan. Specifically, we explore several applications of the LSRRM, which include: (a) detecting individuals whose responses deviate from model expectations, (b) explaining rating relational data in conjunction with covariates, and (c) discovering latent cliques within the LSRRM’s latent space. In application (a), we also demonstrate the robustness of the LSRRM when outliers are present in the data. In application (c), we provide possible interpretations regarding the latent cliques.

### 5.1 Participants

The data were collected through the “A Longitudinal Study of High School Student Networks” project conducted by the Institute of Sociology, Academia Sinica in Taiwan, with IRB approval (AS-IRB-HS07-109008). Forty senior high schools in Taiwan were randomly selected, and 25 of them agreed to participate in the project. Thirty 1st-grade classes were further sampled from the 25 schools. Students could choose to participate in the project after giving informed consent. Each class had at least 12 to 54 students participating in this project.

The data were collected in four waves. For this study, we utilized data from the first wave, collected in October 2021, one month after the school year began. In the first wave, eight classes participated in the project, with a 100% attendance rate and a 100% response rate. The students were asked to complete several questionnaires, including the International Personality Item Pool (IPIP)-15, the Learning Motivation subscale of the Revised Learning and Study Strategies Inventory (High School Version), and a questionnaire about their familiarity with each classmate. The data are available at OSF.

Because our goal is to test the applicability of the LSRRM, we used only the complete networks constructed from the eight classes with sample sizes ranging from 31 to 40, as shown in Table 4. We do not analyze the incomplete networks since the reasons for the missing ratings are unknown. However, in a later section 5.5, we randomly delete subsets of the data for the eight networks to examine the LSRRM’s performance when missingness is present.

**Table 4:** Descriptive statistics for the eight classes

	Class id							
Variable	23	25	26	27	30	39	47	48
Sample size	34	34	35	40	31	30	31	32
Area	Center	Center	South	North	South	Center	South	North
# of females	16	18	35	15	18	19	19	18
Average admission score	23.00	24.94	22.46	26.85	20.00	20.00	20.58	20.31
Average economic status	3.06	3.26	2.91	3.50	2.81	3.00	3.06	3.03
Reciprocity	.9110	.8983	.8840	.8797	.8798	.8684	.8970	.8619
Clustering	.7167	.6514	.7021	.6965	.7040	.6872	.7261	.6635

*Note*: Class id: IDs were randomly assigned by the database, starting with the number 20.

### 5.2 Instruments

The following scales were used in this section.

#### 5.2.1 Familiarity

Similar to Gruenfeld et al. (1996), familiarity was measured by asking each student to rate their classmates on a 4-point scale. However, before starting the project, the project team conducted a pilot study on the familiarity networks of students by using the same scale in four additional classes from three schools. One hundred and twenty-eight students provided informed consent. Many students reported that the original 4-point scale was too complex. As a result, the project team reduced the number of options to three for the final study. The three options were as follows: (1) do not know him/her at all, (2) know him/her slightly, and (3) know him/her very well.

#### 5.2.2 IPIP-15

The IPIP-15 is an abbreviated Chinese version of the Big Five Inventory (BFI) (Goldberg, 1992) proposed by Li and Chen (2016). This version contains five subscales: Openness to experience ( 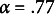 ), conscientiousness ( 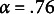 ), extraversion ( 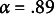 ), agreeableness ( 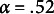 ), and neuroticism ( 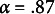 ). Each subscale has three items and is measured on a 5-point Likert scale. Relationships between personality traits and position in a social network have been found in many studies (see Fang et al., 2015 for a literature review).

#### 5.2.3 Learning motivation

Learning motivation is a subscale of the High School Version of the Revised Learning and Study Strategies Inventory (Wu, 2017). This scale contains five items, assessed on a 5-point Likert scale ( 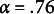 ). The five items include finishing homework on time; finding a way to finish reading, writing, or completing an assignment even if the content is boring; studying hard even if the subject is not liked; studying very hard to get into a better (more prestigious) school; and studying hard to achieve the goals a student sets for her- or himself. We used this scale to examine whether friendship plays an important role in students’ academic motivation (Wentzel et al., 2018).

### 5.3 Descriptive analysis

Taiwan can be divided into four areas: North, Center, South, and East. As shown in Table 4, of the eight classes, two were located in North Taiwan, three were in the center, and three were in South Taiwan. East Taiwan has the fewest senior high schools, so few participated in our project, and none had a 100% attendance rate. Only Class 26 was all female; the other classes had more than 50% females, except for Classes 23 and 27. The admission scores range from ten to thirty, with ten being the lowest, twenty the middle, and thirty the highest score. Classes 25 and 27 had relatively high admission thresholds, with average scores of approximately 27 and 25, respectively. In contrast, the other classes had an average score of 20. For the economic status measure, a score of three indicates an average status. Most classes have average scores slightly above three. Class 27 had the highest status, with an average score of 3.5, while Classes 26 and 30 had relatively low averages of 2.91 and 2.81, respectively.

Class 27 has a higher admission threshold and a better average economic status, which is unsurprising given that North Taiwan has more educational resources. Similarly, Class 25 appears to be a key school in central Taiwan, concentrating on high-achieving students with average economic backgrounds. Nevertheless, this also highlights that families with a stronger economic foundation might have better educational opportunities [ 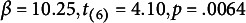 ], consistent with Han et al. (2003)’s findings. However, these factors do not appear to be related to differences among students’ familiarity networks. Instead, the eight networks show strong similarities in the descriptive measures of reciprocity and clustering: The reciprocity indices are greater than 0.85, and the clustering indices are approximately 0.7.

### 5.4 Model analysis

Table 5 presents the Deviance Information Criterion (DIC) values for the different versions of the LSRRM: Euclidean distance, projection distance, and inner product distance. A lower DIC value indicates better model performance. The results show that the LSRRM with Euclidean distance generally provides a better fit for most classes, except for classes 30 and 47. Therefore, we focus on the Euclidean distance version of the LSRRM when presenting the estimation results.

**Table 5:** Deviance Information Criterion (DIC) values of the Euclidean distance, projection distance, and inner product versions of the LSRRM fit to the eight classes

	Class id							
Distance function	23	25	26	27	30	39	47	48
Euclidean distance	1,092.1	1,397.0	1,555.4	1,959.3	1,181.1	1,041.4	1,319.5	1,193.8
Projection distance	1,299.3	1,503.1	1,731.6	2,140.6	1,172.0	1,126.5	1,328.9	1,315.7
Inner product distance	1,295.0	1,434.6	1,703.1	2,084.9	1,066.5	1,076.2	1,255.5	1,292.2

Table 6 summarizes the model parameter estimates. We find that the estimated 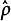 values of the eight classes are positive and high, ranging from 0.5735 to 0.8871. Moreover, they exhibit a strong positive correlation of .8749 [ 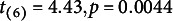 ] with the values of the reciprocity index indicating that 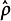 captures the network’s degree of reciprocity. These results show that the eight rated class networks are nearly symmetrical: Students rated each other similarly on the familiarity scale. We also note that 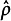 makes finer distinctions among the classes, suggesting that it is a more sensitive measure of reciprocity than Squartini et al. (2013)’s index. For example, according to a Wald-test, the 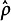 values of classes 23 and 48 are significantly different [ 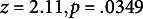 ]. No comparable test for the reciprocity index is available.

**Table 6:** A summary table of the estimates of 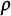 , 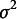 , 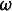 , and 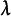 of the LSRRM for the eight classes

	Class id							
Estimate	23	25	26	27	30	39	47	48
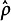 (SD)	.8871 (0.05)	.8303 (0.07)	.7533 (0.08)	.8073 (0.07)	.8163 (0.08)	.6889 (0.11)	.8193 (0.08)	.5735 (0.14)
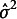 (SD)	4.16 (1.29)	2.59 (0.76)	3.40 (0.92)	3.38 (0.86)	5.00 (1.55)	4.15 (1.18)	3.34 (0.99)	3.11 (0.97)
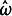 (SD)	.6684 (0.23)	.6686 (0.23)	.6690 (0.23)	.6642 (0.23)	.6647 (0.24)	.6640 (0.23)	.6695 (0.23)	.6608 (0.24)
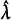 (SD)	2.96 (0.34)	2.20 (0.26)	2.13 (0.24)	2.35 (0.25)	2.72 (0.34)	2.15 (0.28)	1.86 (0.25)	1.98 (0.26)

*Note*: SD: standard deviation.

Additionally, we find that the estimates of 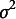 vary from 2.59 to 5 across classes, suggesting that the distributions of sender and receiver parameters are not homogeneous among the eight classes.

The 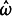 values of the eight classes are greater than 0.5, indicating that the fitted models were influenced by conditional dependence. The estimated 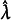 values range from 1.86 to 2.96 and the corresponding clustering indices range from 0.6514 to 0.7261, indicating a strong level of homophily. Again, we find differences between classes. For example, according to a Wald-test, the difference between classes 23’s and 47’s 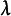 is significant [ 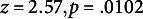 ].

We move now to three applications of the LSRRM that are of interest in applied work. First, we discuss how to detect individuals whose responses are not fitted well by the LSRRM. Second, we include covariates when estimating the person parameters. And, third, we discuss methods that help identify and interpret latent cliques within the LSRRM’s latent space.

#### 5.4.1 Detecting individuals whose responses deviate from model expectations

To assess the fit of the LSRRM, we propose to examine whether the “sent” or “received” ratings deviate from model expectations. If the LSRRM effectively explains the data, we expect that the majority of the responses are predicted well. To assess the fit of the responses, we adapt Glas and Meijer (2003)’s person fit approach under a Bayesian framework. Specifically, sender fit refers to the degree to which the giving ratings deviate from model expectations, and receiver fit refers to the degree to which the received ratings are inconsistent with model expectations.

The sender fit for individual *i* and receiver fit for individual *j* can be calculated as follows: 

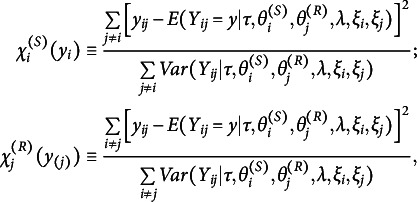

where 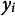 is individual *i*’s ratings, 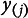 is individual *j*’s received ratings, and 

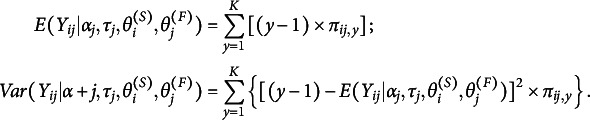



Sender and receiver fit are evaluated by a posterior predictive check: 

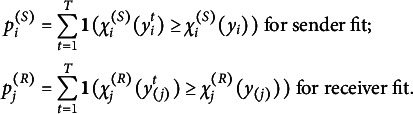

where 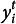 and 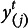 are generated from the posterior distribution for 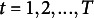 . In this study, we choose 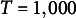 .

For example, in Class 25, the sender fit statistic 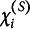 s ranges from 0.72 to 3.72 (M=1.90, SD=0.58). The corresponding 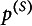 s range from 0.0060 to 0.9890 (M=0.2976, SD=0.2229). The receiver fit statistics range from 0.84 to 3.07 (M=1.88, SD=0.51). The corresponding 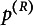 s range from 0.0327 to 0.7547 (M=0.3124, SD=0.2190). Significant model deviations may be diagnosed when 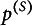 or 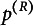 are less than 0.05.

**Figure 4:**
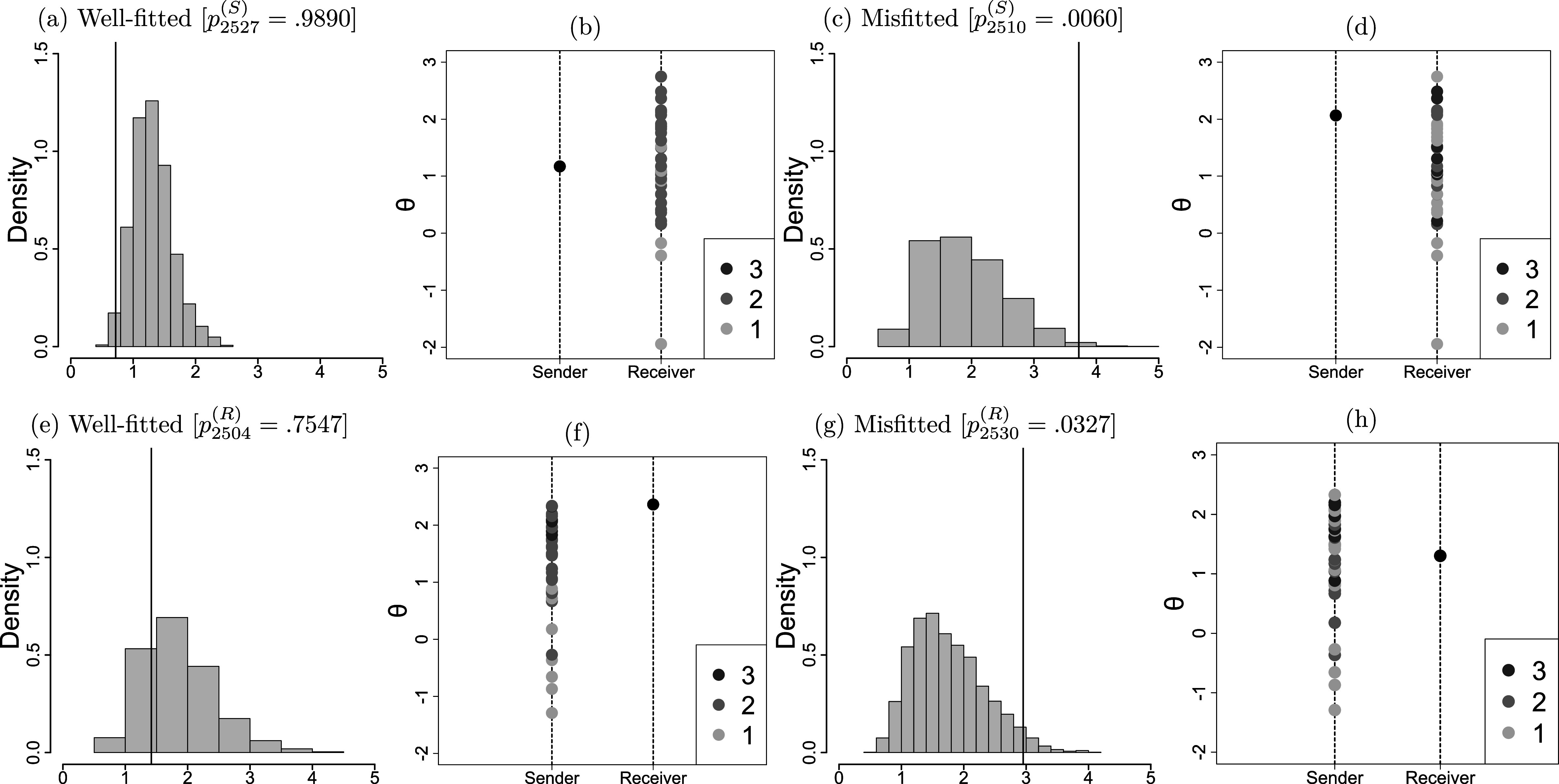
Posterior predictive checks.*Note:* Subfigures (a)(c)(e)(g) plot the samples from the posterior predictive distribution. (b)(d)(f)(h) plot the one-dimensional scatter plots.

To illustrate the sender fit, we use student 2527 (seat number 27, class 25) and student 2510 (seat number 10, class 25) as examples. We arranged students 2527’s and 2510’s ratings to receivers based on the level of receiver parameters, as illustrated in subfigures (b) and (d) of Figure 4, respectively, with darker colors indicating higher scores. These one-dimensional plots allow the sender or receiver parameters to be compared on the same scale. Regarding subfigure (b), the color depth order was almost the same as the size order of the receiver parameters, indicating that most of the ratings of student 2527 were consistent with model expectations. This can also be seen in subfigure (a) of Figure 4, which plots the samples from the posterior predictive distribution, with 
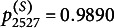
 . In contrast, most of student 2510’s ratings and the level of receiver parameters were inconsistent, as shown in subfigure (d) and determined by subfigure (c) of Figure 4 with 
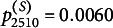
 .

Similarly, to illustrate receiver fit 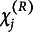 s, we take students 2504 (seat number four, class 25) and 2530 (seat number 30, class 25) as examples. The ratings received by students 2504 and 2530 are arranged on a line according to the level of the sender parameters, as shown in subfigures (f) and (h) of Figure 4, respectively. The order of the received ratings agrees closely with the size of the sender parameters, indicating that the ratings received by student 2504 are almost consistent with model expectations. This result is also reflected in subfigure (e) of Figure 4 with 
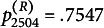
 . On the other hand, the ratings received by student 2530 do not agree closely with the size oderings of the sender parameters, as depicted in subfigure (h) of Figure 4. This suggests that the ratings received by student 2530 are not in line with model expectations. These results are also evident in subfigure (g) of Figure 4 with 
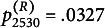
 .

After removing the three individuals with unexpected ratings or unexpected received ratings, we refitted the LSRRM to the data to assess the robustness of the remaining sender and receiver parameters. The results of the paired t-test indicated that there are no significant differences in the sender and receiver parameters before and after excluding the questionable individuals, with [ 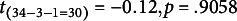 ] for sender parameters and [ 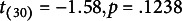 ] for receiver parameters. We conclude that in this application, model predictions are robust even when some individuals exhibit misfit.

#### 5.4.2 Modeling relational ratings with covariates

When covariates are available, interpretation of the model results is greatly facilitated when covariates are incorporated into the LSRRM; that is, 
(9)

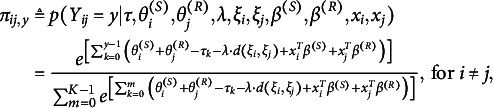

where 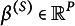 and 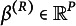 are *P*-dimensional regression weights of the sender *i*’s and receiver *j*’s covariates, 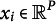 and 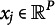 , respectively.

To illustrate this approach, we revisit the data of Class 39 and include gender, the five personality traits of IPIP-15, and learning motivation as covariates. The DIC value reduced from 1,041.41 to 897.20, demonstrating that the covariates improved the fit of the LSRRM.

The covariates were divided into two parts: one for senders and one for receivers. According to the 95% credible intervals (CIs) of the estimated regression weights, significant effects are obtained for senders’ gender (female) [ 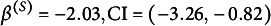 ], senders’ agreeableness [ 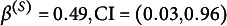 ], and senders’ neuroticism [ 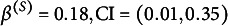 ]. Several of the remaining variables showed marginally significant effects. We list them because of the small network size: Receivers’ learning motivation [ 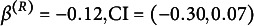 ], and receivers’ neuroticism [ 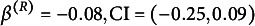 ]. We conclude that the relational ratings for familiarity among the students in this class are associated with the students’ personalities.

#### 5.4.3 Discovering latent cliques

**Figure 5:**
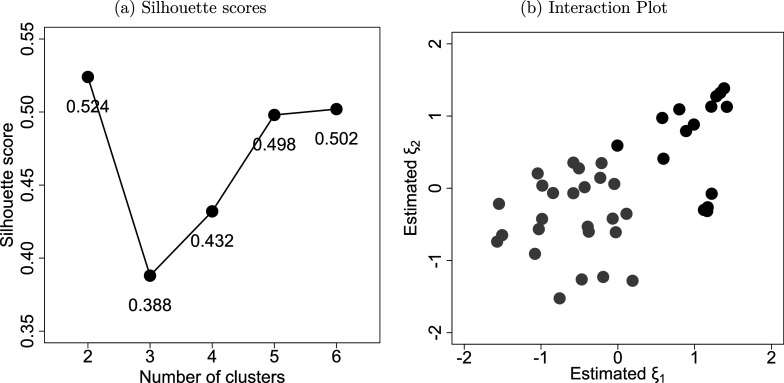
Clustering analysis with the LSRRM.

The respondents’ latent-space coordinates may inform us of unobserved cliques in a sample. To illustrate, we submit the Class-27 students’ two-dimensional latent positions to a k-means analysis. Figure 5 displays the Silhouette scores of the clustering results. The optimal number of clusters is 2, suggesting that the students’ latent positions could be assigned to two groups (sample size: black: 14, red: 26). Subsequent analyses showed that these two groups differ in terms of agreeableness on the IPIP-15 (M: black: 12.29, red: 10.31) [ 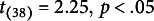 ] and learning motivation (M: black: 20.43, red: 18.04) [ 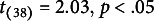 ]. Thus, the students in Class 27 can be categorized into two groups, with one showing higher agreeableness and greater academic motivation.

### 5.5 The effect of missing data on estimation accuracy

Collecting complete network data may be challenging, especially when the group of receivers is large. This raises the question of how accurately the LSSRM can estimate model parameters when only a subsample of the data are available. We will investigate this question with a simulation study.

Subsets were created by randomly selecting a percentage of the ratings provided by each sender. The selected percentages were 15%, 30%, and 50%. The sample size for the 50% case was approximately 15, which corresponds to the scenario with 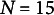 in the simulation study. Next, we applied the LSRRM to the subsample and simulated “new” data using the estimated parameters. To control for sampling error in this step, we repeated the simulation of “new” data 50 times. Finally, we compared the estimated sender and receiver parameters as well as the reciprocity and clustering indices of the “new” simulated network to those of the real data. The results are summarized in Table 7.

From Table 7, it is evident that the sender and receiver parameters are underestimated when using a subsample compared to the estimates obtained from the complete networks. As expected, the estimated error decreases as the sampling percentage increases. For instance, if each sender is randomly assigned 15% of the receivers, the absolute average estimated bias and average RMSE values for the sender and receiver parameters are substantial. However, when the sampling percentage is increased to 50%, the absolute average bias values and average RMSE values reduce by approximately 80% and 60%, respectively.

**Table 7:** Indices assessing the recovery ability of the LSRRM

	15%											
Class id	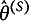			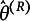			Reciprocity			Clustering		
	Bias	RMSE	Corr	Bias	RMSE	Corr	Value	Bias	RMSE	Value	Bias	RMSE
23	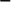 1.24	1.49	.88	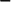 1.24	1.47	.82	.91	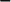 .08	.09	.72	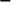 .02	.05
25	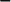 1.11	1.27	.77	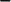 1.11	1.25	.76	.90	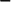 .07	.07	.65	.04	.05
26	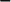 1.35	1.46	.87	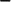 1.34	1.42	.73	.88	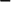 .06	.06	.70	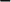 .00	.02
27	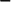 1.15	1.41	.87	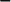 1.16	1.32	.76	.88	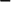 .06	.06	.70	.02	.03
30	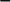 1.67	1.78	.83	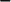 1.67	1.76	.70	.88	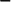 .07	.07	.70	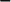 .03	.04
39	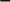 1.32	1.64	.94	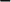 1.32	1.48	.80	.87	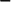 .04	.04	.69	.01	.03
47	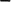 1.50	1.60	.85	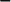 1.50	1.60	.67	.90	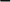 .05	.05	.73	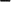 .03	.03
48	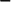 1.12	1.35	.89	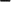 1.12	1.33	.82	.86	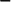 .01	.02	.66	.01	.02
	30%											
23	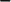 0.34	0.85	.91	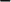 0.34	0.80	.86	.91	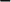 .03	.03	.72	.02	.03
25	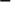 0.59	0.83	.89	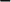 0.59	0.83	.88	.90	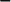 .04	.05	.65	.04	.05
26	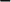 0.92	1.09	.92	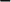 0.92	1.05	.81	.88	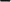 .04	.04	.70	.03	.04
27	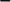 0.30	0.72	.95	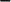 0.30	0.70	.87	.88	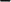 .02	.02	.70	.05	.06
30	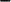 0.98	1.26	.92	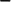 0.98	1.23	.83	.88	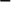 .04	.05	.70	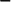 .01	.03
39	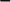 0.64	1.06	.96	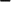 0.63	0.96	.85	.87	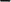 .02	.02	.69	.06	.06
47	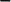 1.49	1.54	.88	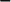 1.49	1.54	.70	.90	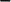 .05	.05	.73	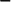 .02	.03
48	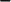 0.99	1.18	.89	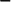 0.99	1.15	.87	.86	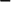 .01	.02	.66	.02	.02
	50%											
23	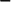 0.17	0.59	.95	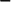 0.17	0.56	.93	.91	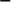 .03	.03	.72	.01	.02
25	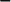 0.16	0.48	.97	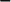 0.16	0.49	.97	.90	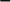 .04	.04	.65	.03	.03
26	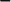 0.32	0.61	.97	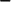 0.32	0.58	.93	.88	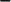 .03	.03	.70	.02	.03
27	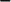 0.10	0.53	.97	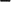 0.10	0.54	.93	.88	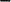 .02	.02	.70	.04	.04
30	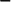 0.39	0.74	.97	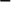 0.39	0.70	.93	.88	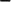 .03	.03	.70	.00	.01
39	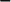 0.21	0.61	.99	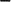 0.20	0.58	.95	.87	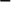 .01	.01	.69	.05	.05
47	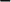 0.56	0.78	.94	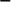 0.56	0.79	.84	.90	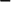 .02	.02	.73	.05	.05
48	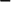 0.38	0.64	.97	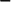 0.37	0.64	.97	.86	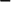 .01	.01	.66	.01	.02

*Note*: Corr: the correlation of estimated parameters between subsample and real data. The bias and RMSE values of 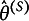 and 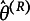 are averaged for summarization.

The correlations between the estimated parameters of the subsample and those of the real data improve as the sampling percentage increases. When 15% of the observations are selected, most of the correlation coefficients of 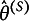 are greater than 0.85. When 30% are selected, these coefficients increase further to over 0.90. However, most correlation coefficients of 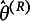 are less than 0.80 in the case of 15% and only increase to more than 0.80 in the case of 30%. This result suggests that although a moderate bias level for 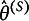 and 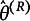 is unavoidable, the precision in estimating 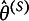 s and 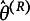 s is modest. This result is caused by our sampling process. While the sampling design controlled for the number of sender ratings, it did not control for the number of receiver ratings. As a result, some receivers may not have received enough ratings to assess their parameters accurately. The impact of this sampling bias becomes less severe when the sampling percentage increases. In particular, the differences in the correlation coefficients are much reduced at a 50% sampling rate.

In summary, we find that the LSRRM is informative about network characteristics when sampling 15% of receivers (about five receivers) from each sender. In this case, the network’s reciprocity and clustering indices from the estimated parameters closely match those of the real data. However, if researchers intend to use the latent traits for further analyses, sampling 30% of receivers (about ten receivers) for each sender is the minimum requirement, consistent with the work of Peng et al. (2023). Sampling 50% (about 15 receivers) provides even more stability, which also corresponds to the sample size we used in the simulation study of complete networks.

## 6 Discussion and conclusion

Relational data comprise ordinal ratings between senders and receivers, resulting in a rated networks. To model these relational data, we introduced several item response models. The most complex of the proposed models, LSRRM, captures both dyadic relationships and unobserved interactions among individuals. This combination allows for a comparison of individuals not only on a one-dimensional latent scale for dyadic relationships but also in a low-dimensional latent metric space for homophily. We also introduced special cases of the LSRRM that are suitable when conditional dependencies are of little importance (DR.RSM) and when binary instead of ordinal data are available (DR.Rasch with and without a latent space component). These models complement approaches developed for “ranked networks” where senders assign a distinct rank to each receiver. In these networks, ranks can be compared within the same sender, but they cannot be compared across senders (Krivitsky & Butts, 2017).

As shown in both simulation and empirical studies, the proposed approach is well-suited to capture such key network properties as reciprocity and clustering. The estimated latent sender and receiver parameters can be compared not only on the same one-dimensional scale for dyadic relationships but also in a low-dimensional space for homophily. For instance, in our application, students rated their familiarity with others in the class. This information allowed us to compare the expressed and received ratings, possibly using covariates, and to examine the data for unobserved cliques. Importantly, model misfit can be assessed at both the sender and receiver levels.

We evaluated the LSRRM’s capacity to capture reciprocity and clustering characteristics of a network in simulation studies. We found that the LSRRM may underestimate the presence of clustering when clustering is low and the network size is small, but this bias is alleviated with larger sample sizes. For incomplete networks, we found that a 30% coverage rate of receivers (approximately 10 in our study) for each sender is sufficient to estimate the complete network with acceptable estimation errors.

There are significant opportunities for future research. Below we list three avenues. First, the current study utilized students’ familiarity networks to evaluate the recovery ability of the LSRRM. These networks resemble small-world networks, characterized by high reciprocity and clustering. It is, therefore, of interest to test the applicability of the LSRRM to other types of networks, such as acquaintance networks.

Second, one benefit of IRT is the option to compare individuals across different groups using anchor items and equating methods (Cook & Eignor, 1991). However, in our current study, the applicability of equating may be an issue. For example, if one link is built between two classes in our empirical study, can we use it as an anchor to compare the students’ familiarity between the two classes? While sender and receiver parameters can be utilized for equating, this may not be possible for the latent positions, as only the distances but not the positions are uniquely defined. A solution to this issue would facilitate comparing the sender and receiver parameters across groups and make predictions about the formation of links among groups.

Third, it may also be useful to model the response styles of senders to allow for such factors as social desirability effects (Böckenholt, 2014; Leng et al., 2020) and extreme response tendencies (Jin & Wang, 2014). These modifications will allow the LSRRM to be utilized in a wider range of scenarios. However, this outlook for future work should not distract from the fact that a useful class of IRT models has become accessible to network researchers. We expect and look forward to more applications in the future.

## Figures and Tables

**Listing 1:**
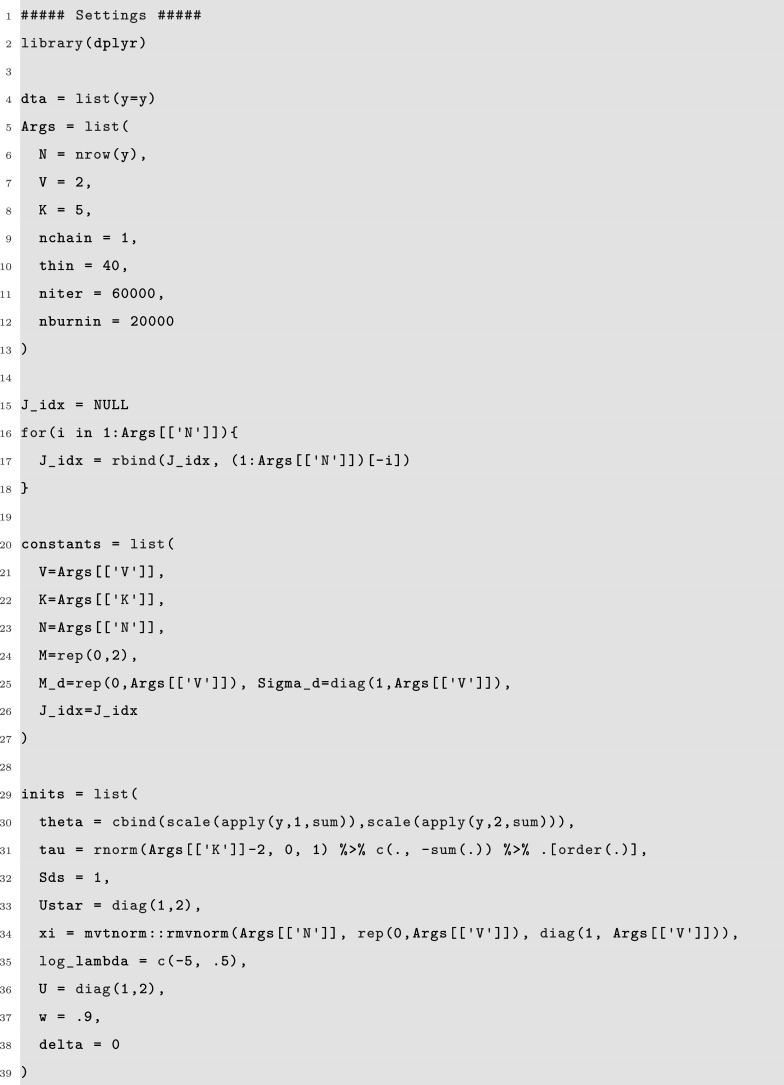
Settings before analyzing.

**Listing 2:**
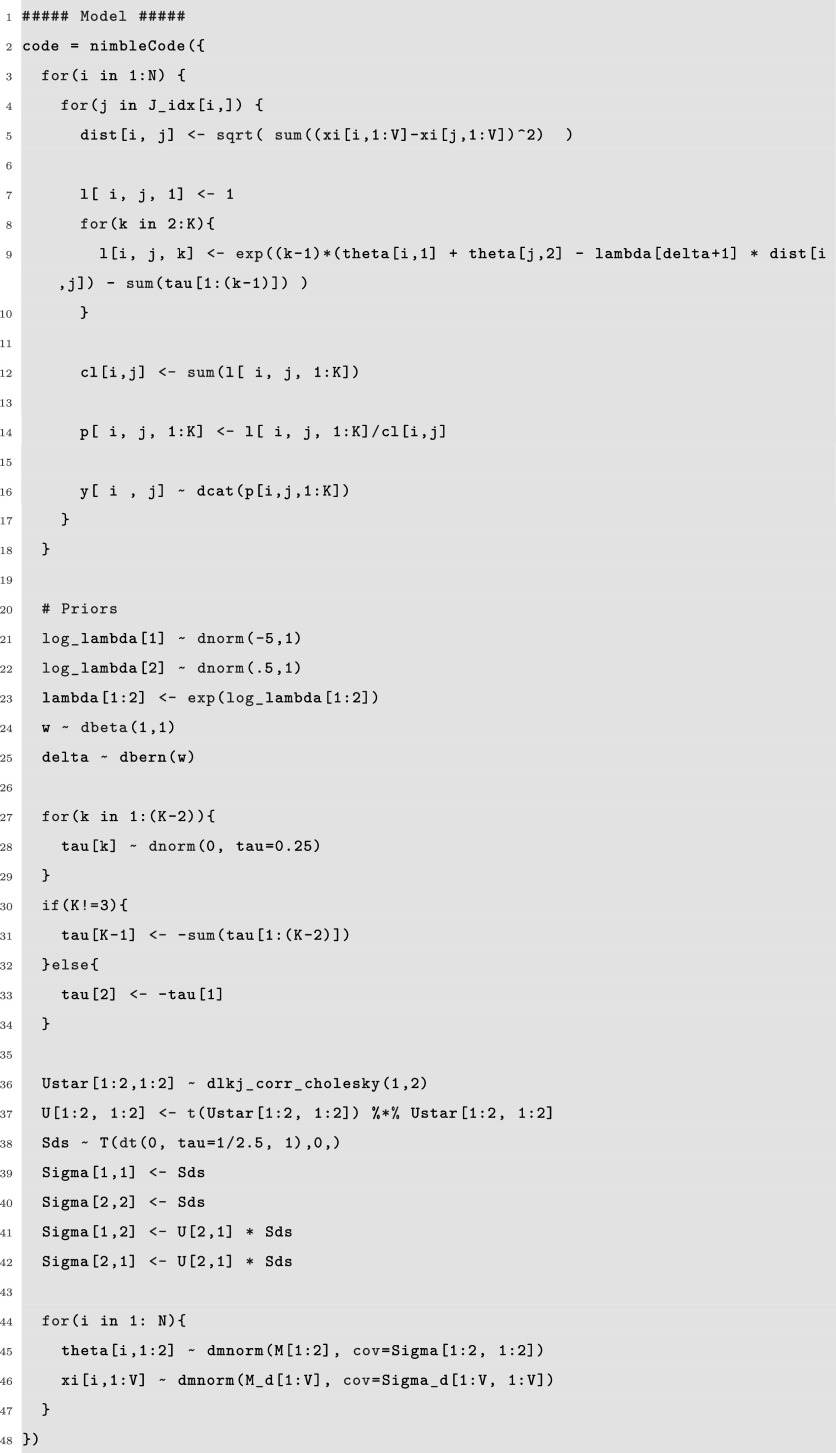
Nimble codes for the LSRRM.

**Listing 3:**
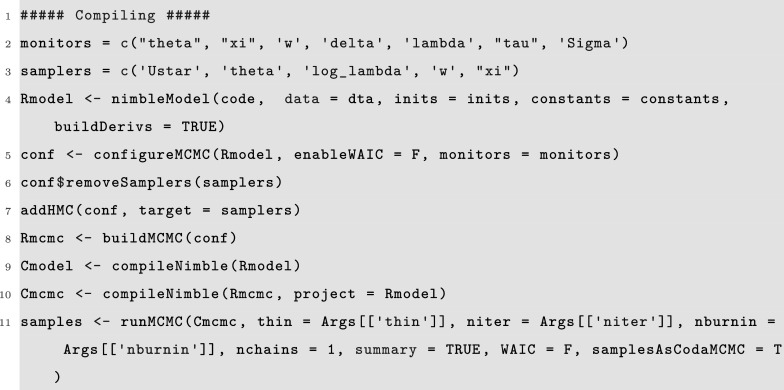
Compiling method for the LSRRM.

**Listing 4:**
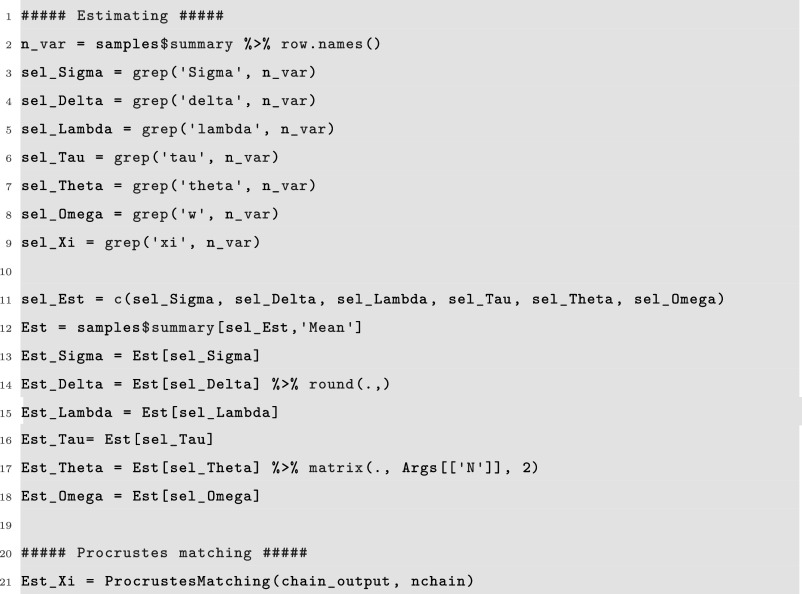
Parameter estimation.

**Listing 5:**
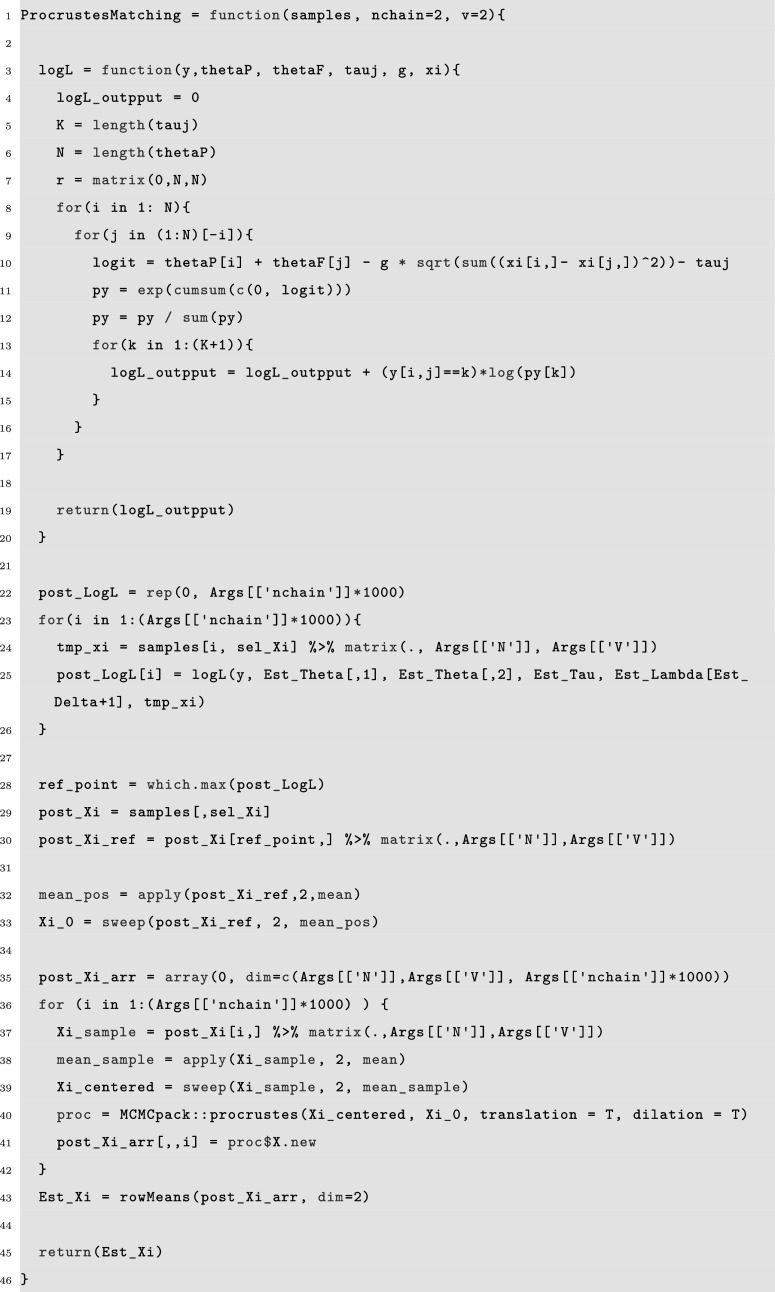
Procrustes matching.

## Data Availability

The R code can be accessed by searching for the “LSRRM” repository on GitHub. The data are available on the open science framework (OSF) at https://osf.io/pqgy4/.

## Appendix A

### A.1 Model estimation in NIMBLE

Before running the estimation code, researchers should prepare their dataset (*y*) and configure the model as demonstrated in Listing 1. Notably, *y* should be organized as an  (adjacency) matrix. *V* denotes the dimensionality of the latent positions  , which is set to two for this study. *K* represents the number of ordinal categories. The term “nchain” refers to the number of chains running sequentially during the process. Additionally, the arguments for thinning (thin), the total number of iterations (niter), and the number of burn-in (nburnin) should be adjusted depending on the convergence of the algrithm. We assess convergence using the  value calculated by the “rhat” function in the “mcmcr” package.

Secondly, the LSRRM is implemented as shown in Listing 2. The hyperparameters for each prior distribution are set as described in Section 3. However, researchers can modify these hyperparameter settings as needed. The coded model is adaptable to any configuration of *V* and *K*, allowing researchers to use it directly for their analyses.

We use Listing 3 to compile the nimble codes for the LSRRM. The term “monitors” refers to the parameters whose posterior distributions researchers are interested in, and these will be output at the end of the process. Here, chains run sequentially during the procedure. For parallel processing, the packages “foreach,” “parallel,” and “doParallel” can be utilized. Additionally, the parallel processing code version is available on GitHub.

Finally, model parameters can be estimated using the EAP method, as shown in Listing 4. In this context, we estimate the parameters  ,  ,  ,  ,  , and  . Additionally, as discussed in Section 2, since  is unidentifiable, we post-process its posterior samples and align them with the reference set using Procrustes matching. The function for Procrustes matching is illustrated in Listing 5.

In the Procrustes matching process, we select the sample of  that has the largest log-likelihood value as the reference set given the estimates of the other parameters. Therefore, before proceeding with the matching, we calculate the log-likelihood value for each posterior sample of  and choose the one with the highest value as the reference set. Next, we use the “procruste” function from the “MCMCpack” package for Procrustes matching. This process enables us to obtain the estimates of  , as shown in Listing 5.

## Appendix B

### B.1 Numerical study of the relation between and r(Y)

To assess the correlation between  and  , four conditions were tested, combining two parameter variations:  and  . For each condition and using the LSRRM, we simulated 1,000 datasets with a sample size of 15 and a category number of five with

a fixed  , and a fixed  . Next, we computed the Pearson correlation between the generated  s and the reciprocity index of the simulated datasets. When  , the correlation coefficient between  and  is 0.8402 [  ] and 0.9031 [  ] for  and  , respectively. When  , the correlation coefficient between  and  is 0.8421 [  ] and 0.9240 [  ] for  and  , respectively. These results indicate that the correlation between  and  is mostly a function of  but also weakly positively moderated by  .

### B.2 **Numerical study of the relation between** **and r(Y)**

To assess the correlation between  and  , we simulated 1,000 datasets with a sample size of 15, a category number of five with  , a fixed  , a fixed  , and a fixed  . Next, we computed the Pearson correlation between the generated  s and the clustering index of the simulated datasets. The result indicates that the Pearson correlation coefficient between  and  is  0.9630 [  ], showing a strong negative relationship.
